# How generativity affects organic dining intention: Case study of Shanghai

**DOI:** 10.3389/fpsyg.2022.1012550

**Published:** 2022-09-15

**Authors:** Yu Pan, Jian Ming Luo, Jiajia Xu

**Affiliations:** ^1^Faculty of International Tourism and Management, City University of Macau, Macau, Macau SAR, China; ^2^Department of Foreign Language, Fuzhou University of International Studies and Trade, Fuzhou, Fujian, China

**Keywords:** generativity, environment concerns, attitude, dining intention, organic food

## Abstract

With people’s concerns about the environment growing, the demand for organic food has increased. However, few studies have focused on organic dining intention. Therefore, this study examined generativity, which is defined as the direction and care for the growth of future generations through self-expanding forms, and its influence on attitude toward organic food, environment concern and dining intention. The moderating effect of age was also examined. A total of 418 responses were collected through a face-to-face survey from Shanghai respondents. PLS-SEM was utilized to verify the model and test the relationships among the constructs. The results show that generativity directly influences environmental concerns, attitudes toward organic food and dining intentions. Furthermore, environmental concerns and attitudes toward organic food are mediating variables for generativity to affect dining intentions. The older the age group, the more likely it is for generativity to have a positive influence on attitude toward organic food and environmental concerns. Theoretical and practical implications are also discussed in this study.

## Introduction

The reckless exploitation and consumption over the years resulted the natural resources’ depletion and serious environmental problems (Bengtsson et al., 2018). Environmental issues have become an increasingly important topic in business and academia, which enhances the concept of sustainable development. Sustainable development encourages ecological innovation and advocates green consumption (Joshi and Rahman, 2015). Green consumption is critical as consumers would prevent and reduce the damage to the natural environment (Moisander, 2007). Organic foods provide an agricultural production with pro-environmental. As a result of the growing number of green consumers, there is a huge increase in demand for organic food. Previous studies have widely studies regarding organic food (Xie et al., 2015; Hansen et al., 2018; Kushwah et al., 2019b; Feil et al., 2020; Rana and Paul, 2020). However, little studies focused on consumers’ dining intentions in the context of organic restaurants. Consumers not only buy organic food in markets, but also consume it in organic restaurants. To meet this growing demand, this study focused on assessing the consumers’ organic dining behavior in restaurant from an environmental perspective.

Consumers’ attention to the environmental issues and their responsibility were found to have a positive impact on green consumption (Makatouni, 2002). Environmental concerns are related to an individual’s moral or ethical obligations (value; Doran, 2009) and contributes to the consumption trend of organic food (Padel and Foster, 2005; Joshi and Rahman, 2015). According to Lian and Yoong (2019), one of the primary motives for choosing organic products is environmental concerns. The range of psychological variables associated with pro-environmental behavior in previous studies is broad. Attitude toward organic food is an important predictor of promoting green consumer behavior (Pham et al., 2019; Ahmed et al., 2021). Individual psychological factors influence how people view the world, while some states may persist longer under various conditions. People with lasting behavior play a significant part in environmental degradation and recovery, with long-term consequences for themselves and future generations (Afridi Ahmad et al., 2021b). This brings up the problem of intergenerational justice, which is often overlooked in marketing and other business-related professions (Afridi Ahmad et al., 2021b). To fill this gap, this study introduces the concept of generativity into this research topic.

Generativity (GEN) is a psychological variable which represents a person’s future orientation (Erikson, 1950b). Because of its connection to intergenerational justice, it is worth investigating further to explore its function in green purchase behavior in depth. According to Afridi Ahmad et al. (2021a), individuals with higher generativity stay deeply invested in social cohesion, ecological system repair and the safety of future generations. Previous studies explored the relationship among generativity, consumers’ attitudes and purchasing intentions toward generatively positioned products (Lacroix and Jolibert, 2017; Luo and Ren, 2020; Luo and Ye, 2020; Fan and Luo, 2022). Exploring the critical role of generativity on green consumer behavior enhances the benefits of studying the possible influences of present actions on the future (Afridi Ahmad et al., 2021b). For organic food consumption, recent research has indicated that environment concerns reflect maintained benefits for future generations (Kapuge, 2016). In this sense, generativity would be crucial in organic food consumption, since consumers may buy organic food to meet their generative requirements.

Therefore, generativity value is introduced in this study and delves into the function in organic food dining behavior. Because generativity is critical to people in later stages (Erikson, 1950b), the moderating effect of age was also considered in the theoretical model. This study based on the Value-Attitude-Behavior model, combined with environmental concerns, investigates the relationship between generativity and green purchase behavior. This study aims to fill the gap by investigating how generativity influence organic dining intention. Meanwhile, this study adds to the literature on the psychosocial attributes of generativity on green purchase behavior in the hospitality industry. The theoretical contributions of this study can provide management insights and recommendations for practitioners of organic restaurants or other green marketing. Thus, our research objectives are as follows:

1. Explore how generativity results in customers’ attitude toward organic food and environmental concerns.
2. Examine how the customers’ environmental concerns affect their attitude toward organic food and how customers’ attitude toward organic food and environmental concerns affects their organic dining intention.
3. Examine the age positively moderator role in generativity to environment concerns, attitude, and dining intention, respectively.

This study examined the positive effects of generativity on attitudes toward organic food, environmental concerns and dining intention. The positive moderating role of age in generativity on attitudes and environmental concerns was verified, respectively. Furthermore, this study verified the mediating role of environmental concerns in the framework. The findings of this study contribute to a deeper knowledge of how customers in organic restaurant consuming situations are impacted by their innate generativity values, affecting environmental concerns and attitudes and providing insights for new green behaviors.

## Literature review

### Organic food and organic restaurant

According to the Standardization Administration of the People’s Republic of China, with specific organic production principles (GB/T 19630–2019), no synthetic pesticides, fertilizers, growth regulators, feed additives, or other substances are used in organic production such as plant production, wild collection, edible fungus cultivation, livestock & poultry breeding, aquaculture, and bee breeding. The balance between cultivation and farming is harmonized in accordance with the laws of nature and ecology, and even artificial materials used in organic food must be approved by the organization (SAPRC, 2019). The organic food production process requires more input and attention from farmers and regulators than the conventional food production process. Over 350 hectares of agricultural land are used for organic food production in China (Peng, 2019). The government intends to invest around 191.2 million USD on new farmers training, with an emphasis on organic and sustainable agriculture (IFOAM, 2016).

The emerging middle class urban consumers who are increasingly concerned about health and environmental issues, influenced by global lifestyle trends, are the main target group for organic food (Deliana, 2012). Research has shown that environmental awareness and environmental conscious consumers are more likely to eat organic food (Saleki et al., 2019; Ahmed et al., 2021). In 2020, organic food sales reached 75.2 billion USD in China (Ma, 2021), and is expected to continue growing in the future. Organic food has received wide attention, which has also affected the restaurant industry. Not only can consumers buy organic food in the market, but organic restaurants are also an option. The ingredients in organic restaurants all fulfil the requirements of organic food. Organic dishes have the least amount of artificially modified ingredients, such as chemical/synthetic seasonings, as well as artificial flavors and colors (Zhang, 2016). For example, Shanghai Shijia Catering Co., Ltd., Zhenggu Kitchen (Beijing) Catering Management Co., Ltd. and Shanghai Dori Agricultural Development Co., Ltd. became the first batch of certified companies (Zhang, 2016). The growth of the number of restaurants reflects the increased public interest in organic food. Consumer demand for organic restaurants is expected to rise. Scholars argue that the organic restaurant consumption trend is rooted not just in taste and health but also in public interest in environment (Kang et al., 2015; de Magistris and Gracia, 2016). Organic food consumption reflects consumers’ concern for the environment, as organic food during planting without biotechnology, such as herbicides and pesticides, not only reduces the risk of food safety for consumers, but also greatly reduces pollution of soil and rivers (Mondelaers et al., 2009). Furthermore, the organic products consumption is related to specific value systems that may influence personality, attitudes and consumption behaviors (Schifferstein and Ophuis, 1998).

Studies regarding consumer behavior of the organic restaurants are summarized in Table 1. Previous studies confirmed that environmental concerns and health consciousness are the main factors driving consumers to select organic food in restaurants (Jeong and Jang, 2019; Shin et al., 2019; Shin and Mattila, 2019). Therefore, the consumers’ lifestyles have intentionally evolved in tandem with this trend. The reason behind this phenomenon may stem from their values. For this reason, further research on organic restaurant intentions is necessary. This study focuses on the impact of consumers’ values on their environment concerns, attitudes, and organic dining intention.

**Table 1:** Research of organic restaurant.

Literature	Purpose	Results
Hong and Ahn (2021)	To test the influence of signaling social status regarding organic restaurant on consumers identification, evaluation, and revisit intention of organic restaurant brand	Signaling social status positively direct influence on identification, evaluation, which in turn have a positive indirect influence on revisit intention.
Konuk (2019)	To examine the effect of perceived food quality, price fairness, value, and satisfaction on the WoM and revisit intention	Satisfaction mediated the relationship between intention and perceived value, food quality as well as price fairness
Shin and Mattila (2019)	To explore the joint influence among initial food choice, gender, and health consciousness on organic food choice	Under the condition of low-level health consciousness, compared with female, initial choice is more relevant with male subsequent food choices.
Jeong and Jang (2019)	To test the moderating effect of consumers characteristics	Gender, age, and health consciousness moderating the influence of premium price on organic menu paying intention.
Shin et al. (2019)	To identify the motivations of intention to organic restaurants and its influence on willingness to pay	Environment concerns, social value, as well as health consciousness are three key motivations, which positively influence on willingness to pay.
Lu and Chi (2018)	To test the influence of perceived value on organic dining intention	Hedonic and utilitarian value influence dining intention through satisfaction.
Shin et al. (2018)	To explore the consumer organic choice intention based on theory of planned behavior (TPB).	TPB model explained well on organic food menu choice intention
Shin et al. (2017)	To examine the relationship among sustainability values, pro-environmental attitude, and organic food menu choice intention	Sustainability values positively influence choice intention through pro-environmental attitude
Hanks and Mattila (2016)	To explore the mechanism of offering organic menus benefits for purveyors	Offering organic menu could enhance consumers perception of corporate social responsibility and trust, which in turn, influence their behavioral intention.

## Generativity, attitude toward organic food and dining intention

In social psychology research, the Value-Attitude-Behavior (VAB) model is widely used to understand behaviors (Kang et al., 2015). In researching green marketing, Homer and Kahle (1988) indicated a causal relationship from abstract cognition (values) to moderate cognition (attitudes) to concrete behavior. Personal values may influence the consumers’ attitudes, which can affect certain behaviors. The hierarchical influence of Value-Attitude-Behavior was used in various studies: explaining consumer recycling behavior (McCarty and Shrum, 1994), pro-environmental behavior (do Paço et al., 2013), and consumers’ healthy food choices at restaurants (Kang et al., 2015). Following the VAB model, the distinct context of the research illustrates the consistent relationships among value, attitude, and behavior.

The values of a person are used to evaluate events and select actions, as well as to rank such events and acts in order of perceived importance (Homer and Kahle, 1988). Generativity is an expression of psychosocial maturity in adulthood (McAdams et al., 1993). People’s resources are devoted to maintaining and enhancing the next generation as they age. In adulthood, developmental expectations about contributing to the next generation and inner impulses for agential immortality and communal nourishment combine to boost the amount to which the individual cares about the development of the next generation. Generativity embodies the human value of belief in a better future, when adults who lack this belief may have difficulty committing to generative action (McAdams and de St Aubin, 1992). The value of the concept of generativity is utilized in environmental studies because it necessitates that the requirements of the current generation be addressed without compromising the ability of future generations to meet their needs and improve well-being for the future generation (do Paço et al., 2013). In this sense, it is closely related to the concept of sustainable development. Lacroix and Jolibert (2017) showed that individuals with higher generativity are also aware of and concerned about the environment, which leads to behavioral changes.

Ajzen and Fishbein (2000) explained attitudes are formed immediately after receiving any product attribute information, and consumers promptly subscribe the value of this attribute to the object under consideration. Existing research has found that perceived health is a core qualitative attribute that positively influences diner attitudes and behavioral intentions (Hur and Jang, 2015). In addition to promoting healthy eating behaviors, sourcing organic food represents a restaurant’s participation in green practices (Wang et al., 2013; Lu and Gursoy, 2017), which can also promote consumer attitudes and behavioral intentions (Jeong et al., 2014). Previous research on organic food has confirmed that consumer attitudes positively influence purchase intentions (Pham et al., 2019; Ahmed et al., 2021). Through a review of the relevant studies, attitudes toward organic food attributes such as taste, health, food safety, environmental friendliness have been recognized as critical conditions for facilitating consumer decisions about organic food consumption (Bryła, 2016). Attitudes are generally important predictors of behavior. According to Ajzen and Fishbein (2000), a person is more likely to engage in a behavior if he/she has a positive attitude toward it.

Behavioral intention is a direct determinant of behavior (Fishbein and Ajzen, 1977). This means that accurate measurement of behavioral intention allows for a comprehensive understanding of behavior. Therefore, this study uses consumers’ intention to visit an organic restaurant as a proxy for possible behaviors. According to Urien and Kilbourne (2011), individuals with a higher generativity engage in pro-environmental consumption behavior, including buying organic, saving energy and green products. Individuals with a higher generative concern, as previously noted, continue to play an important role in the cohesiveness, rebuilding and care of next generations (Aubin and McAdams, 1995). Most research is ambiguous on how to build a greener environment or the extent to which environmental concerns impact consumer choices. Previous research have explored the positively association with generativity and attitudes toward green products (Afridi Ahmad et al., 2021a; Sharma et al., 2022). Given the increased importance of GEN in examining the possible future effect of current actions, it is worth exploring the importance of GEN in consumer behavioral intentions. Prior studies widely explored positive relationship between generativity and green buying behavior (Shiel et al., 2020; Afridi Ahmad et al., 2021a; Zaidi et al., 2022). Moreover, Empirical research revealed that the generativity of consumers positively associated with their engagement and consuming intention toward organic products (Gani et al., 2022). This study believes that individuals with higher generativity may be more inclined to visit environmentally friendly organic restaurants. Therefore, the following hypotheses are proposed by the study:

*H1*: Generativity positively influence attitude toward organic food.

*H2*: Attitude toward organic food positively influence organic dining intention.

*H3*: Generativity positively influence organic dining intention.

### Environmental concerns

Environmental concerns may be defined as the extent to which individual are aware of environmental challenges and exert efforts to find solutions (Jiang and Kim, 2015). According to Zhao and Chen (2021) research on the green housing setting, consumers with higher environmental concerns would focus on environmental performance of a product. As a result, his concern rises for environmental acts, such as green buying intentions (Bouscasse et al., 2018). Liao et al. (2020) shows that environmental concern is a necessary condition for pro-environmental behavior. Wei et al. (2018) established that customers’ intent to visit a green hotel is influenced by their level of environmental awareness.

The relationships between generativity and consumers’ green purchase behavior has been explored (Urien and Kilbourne, 2011; Afridi Ahmad et al., 2021b). However, the fundamental mechanism underpinning the relationship between GEN and green purchase behavior is unclear. One of the key goals of this study is to confirm the involvement of environment concerns in mediating the connection between generativity and dining intention. Although there is scant evidence linking generativity and environmental concern, researchers have identified generativity as an essential factor to environment concerns (Moore and Nelson, 2010). Pratt et al. (2013) investigated the influence of generativity on environment concerns in adolescents and their parents and found that GEN had a favorable and substantial effect on environment concerns. According to Teng et al. (2014), personal values are positively correlated with macro-level environmental issues. Because the generativity indicates responsibility and concern for future generations, it may better inspire people to care about the environment (Testa et al., 2015). Prior studies investigated generativity positively influence environmental concerns (Sunderman, 2020; Sharma et al., 2022). Therefore, the following hypothesis is proposed by the study:

*H4*: Generativity positively influence environment concerns.

Previous studies have indicated that consumers who are more concerned about the environment are more likely to engage in eco-friendly consumer behavior (Kalafatis et al., 1999; Laroche et al., 2001; do Paço et al., 2013). Testa et al. (2015) reported a strong link between health promotion and environmental concerns, which leads to the purchasing of green products. Organic food is beneficial for the environment since it is devoid of herbicides and chemical pesticides, which is one of the reasons that drive people to consume organic food (Smith and Paladino, 2010; Shafie and Rennie, 2012; Saleki et al., 2019). Empirically, Kushwah et al. (2019a) revealed that consumers’ environmental concerns positively associated with their buying intention of organic food. This positive relationship was also identified in another investigation of young groups (Ahmed et al., 2021). Hu et al. (2010) discovered a significant association between environmental concern and patronage desire to patronize green eateries. Furthermore, Hirsh (2010) discovered that environmental concerns have a strong positive influence on attitudes toward wild fish. Many studies have also proven that environmental concerns may positively influence pro-environmental intention and conduct (Chin et al., 2018). Bech-Larsen (1996) indicated environmental awareness contribution to positive attitude toward certain products. The following two hypotheses are proposed in the study:

*H5*: Environmental concerns positively influence organic food attitude.

*H6*: Environmental concerns positively influence dining intention.

### The moderating effect of age

Previous studies explored the effect of age on environment concerns. Several studies conducted in the 1980s and 1990s indicated that young people were more concerned about environmental problem than older ones (Liere and Dunlap, 1980; Howell and Laska, 1992; Dietz et al., 1998). However, Liu et al. (2014) argued that older individuals in the United States are more concerned about the environment than younger folks. Therefore, environmental concerns of people from various ages may vary. Samarasinghe (2012); Wang et al. (2020), and Molinillo et al. (2021) investigated the role of age in determining the consumer behavior. Additionally, individuals with various ages perceived behavior consequences differently (Kim, 2012). Thus, attitude and behavioral intention of consumers may differ with age. Generativity is crucial to individuals in the later stages of their lifespan, and it is essential to human growth as well as maturity (Erikson, 1950b). The moderator variable can influence the direction of a relationship in the model between two constructs. When there is a positive moderating effect, the more positive moderator is, the more positive the effect of antecedents on consequences becomes (Hair et al., 2021). Basis on the literature review, the study proposes the following hypotheses:

*H7a*: Generativity has a positive effect on attitude, which is positively moderated by age.

*H7b*: Generativity has a positive effect on environment concerns, which are positively moderated by age.

*H7c*: Generativity has a positive effect on dining intention, which is positively moderated by age.

**Figure 1:**
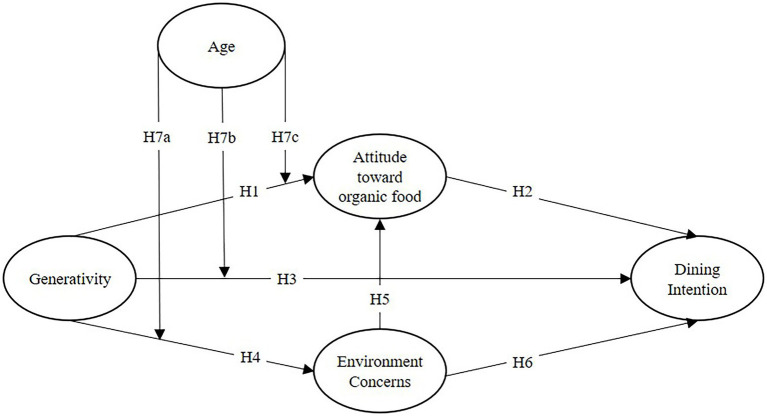
Research model.

The study proposed hypothesis relationship of the constructs are presented in Figure 1. This study used the VAB model with the addition of H2, H3 and H5 as previous literature review (Hirsh, 2010; Afridi Ahmad et al., 2021b). Finally, the study tested the moderation effect of age on H7a, H7b and H7c.

## Methodology

### Research instrument

All measurement scales were adopted from previous studies and a questionnaire was designed. Previous research has shown that all measuring scales employed in this study are reliable and valid. These items were translated from English to Chinese, then revised and translated back into English to assure correctness (Brislin, 1970). Thereafter, the questionnaire was evaluated by 25 students graduated with a degree in hospitality and tourism and updated depending on their comments. Bilingual experts reviewed the measuring scales to confirm that the Chinese and English versions of the questionnaire were equivalent. There are 27 items in part 1 measuring the four constructs while respondent’s demographic information was collected in Part 2. A five-point Likert scale was used to reflected the respondent’s feedback from 5 (“strongly agree”) to 1 (“strongly disagree”).

Generativity: the contribution dimension of generativity was assessed with three items; the remembered dimension of generativity was assessed with five items; the creativity dimension of generativity was assessed with two items; the responsibility dimension was assessed with two items; the knowledge dimension was assessed with three items. Take “I feel as though I have made a difference to many people,” as an example, the generativity scale was created in response to the findings of Urien and Kilbourne (2011) and Wells et al. (2016). The attitude scale was adopted from Shin et al. (2018). Four items were used to measure the customers’ attitude toward organic food. A sample item is, “Unattractive\Attractive.” The scale of environmental concerns adopted from Amalia et al. (2021). Three items were used to measure the customers’ environmental concerns. “I have a concern about the environment,” as an example. The scale of dining intention adopted from Shin et al. (2018). Three items were used to measure dining intention. A sample item is “I am planning to visit an organic restaurant in the future.”

### Data collection

Shanghai is one of the first cities in China to establish organic restaurants. This city has hosted several conferences related to organic food (SNIEC, 2019), and will organize more exhibitions and trade fairs on organic food in the future (IFOAM, 2020). Therefore, our questionnaire was distributed in Shanghai where organic food is gaining more attention. The research subjects were conducted among individuals who were (1) aware of organic food and (2) knew about organic restaurants in Shanghai. In order to identify if a participant belonged to the research subjects, the definition of organic food and the two screening criteria were described before participants began the survey. The organic food is defined as food produced without the addition of chemical fertilizers, herbicides, pesticides and biotechnology (SAPRC, 2019). An organic menu was defined as any restaurant item manufactured partially or entirely with certified organic ingredients. Since this study could not get the personal information of the whole population in Shanghai, this study adopted non-probability sampling methods. Convenience sampling is a common sampling method adopted by studies focused on consumer behavior regarding organic food (Rödiger and Hamm, 2015), which is the least time cost and easiest to implement (Bornstein et al., 2013). Convenience sampling was used in this study form October to November 2021. Professional interviewers conduct face-to-face surveys around Nanjing Rode in Shanghai’s key regions (e.g., apartment stores, retail malls, underground entrance) to collect data. In interviews, Mandarin was the predominant language used. Each interview lasts an average of about 10 min.

### Sample profile

The demographic information of the samples is summarized in Table 2. In terms of age distribution, 100 (24%) individuals were aged 20–29 years, 115 individuals (27%) were aged 30 to 39 years, 122 (30%) were aged 40 to 49 years, and 83 (19%) were aged 50–75 years. Regarding gender, females account for 56%, and males account for 44%. More than half of the respondents were married (60%), and 99% of respondents held a university diploma or above. In terms of occupation, most of the respondents were working (68%), while other respondents were students (25%), housewife (2%), or retired (5%). Compared with the seventh census of Shanghai Shanghai Bureau of Statistics (2021), the female respondents are relatively higher than the male respondents. Regarding age and education, the sample profiles are similar to the census data, with the major respondents aged 20–59 and having bachelor’s degrees or above.

**Table 2:** Demographic profile of respondents (*N* = 418).

**Variable**		**Frequency**	**Percentage**
Gender	Male	186	44%
	Female	232	56%
Marital Status	Married	249	60%
	Single	169	40%
Age	20–29 years old	100	24%
	30–39 years old	114	27%
	40–49 years old	121	30%
	50–59 years old	68	16%
	60–75 years old	15	3%
Education	High school or below	5	1%
	Diploma	47	11%
	Bachelor’s degree	325	78%
	Master or above	41	10%
Occupation	Student	103	25%
	Working	282	68%
	Housewife	11	2%
	Retired	22	5%
Personal Monthly Income (RMB)	Less than 5,000	84	20%
	5,000–9,999	138	33%
	10,000–14,999	105	25%
	15,000–19,999	63	15%
	20,000–29,999	17	4%
	30,000 and above	13	3%

## Findings

Partial least squares structural equation modeling (PLS-SEM) is the second-generation statistical method of multivariate analysis applied by social science researchers, which enable scholars to investigate latent variables measured indirectly by explicit indicators and estimate relationships among latent variables (Hair et al., 2021). SmartPLS 3 is a software to conduct the algorithm of PLS-SEM, which has been adopted in many research fields, including green consumption. In this study, SmartPLS 3.3.3 was utilized to verify the model and test structure relationships among the constructs based on the 418 valid data collected.

### Measurement model evaluation

In this study, the cumulative variance of the single component factor is 44.267 percent, which is less than the 50% (Kock et al., 2021). As a result, the data do not exhibit significant common method bias. Table 3 shows the values of the factor loading, Cronbach’s alpha, average variance extracted (AVE) and construct reliable (CR) of the measurement variables of the research model. The values of Cronbach’s alpha ranged from 0.721 to 0.926, while the CR value ranged from 0.844 to 0.940. According to Hair et al. (2010), the threshold of Cronbach’s alpha for all constructs are higher than 0.7. The value of construct reliable and AVE for all constructs exceed 0.8 and 0.5, respectively, indicating adequate reliability and convergence validity (Hair et al., 2010).

**Table 3:** Item, reliability, and convergence validity.

**Variables & Measured Items**	**Factor Loading**	**Cronbach’s alpha**	**CR**	**AVE**
**Contribution Generativity**		0.721	0.844	0.643
I feel as though I have done nothing of worth to contribute to others.	0.815			
I have a responsibility to improve the neighborhood in which I live.	0.821			
I feel as though my contributions will exist after I die.	0.768			
**Remembered Generativity**		0.861	0.900	0.643
I feel as though I have made a difference to many people.	0.818			
I have made and created things that have had an impact on other people.	0.799			
I think that I will be remembered for a long time after I die.	0.772			
Others would say that I have made unique contributions to society.	0.796			
In general, my actions do not have a positive effect on other people.	0.823			
**Creativity Generativity**		0.758	0.892	0.805
I try to be creative in most things that I do.	0.893			
Other people say that I am a very productive person.	0.902			
**Responsibility Generativity**		0.748	0.888	0.799
I do not feel that other people need me.	0.888			
I have made many commitments to many different kinds of people, groups, and activities in my life.	0.899			
**Knowledge Generativity**		0.832	0.900	0.749
I try to pass along the knowledge I have gained through my experiences.	0.883			
I have important skills that I try to teach others.	0.882			
People come to me for advice.	0.831			
**Attitude Toward Organic Food**		0.919	0.940	0.805
Disadvantageous / Advantageous	0.886			
Foolish / Wise	0.907			
Unpleasant / Pleasant	0.892			
Unattractive / Attractive	0.888			
**Environment Concerns**		0.926	0.935	0.871
I have a concern about the environment	0.927			
For the ecological reason, switching the nonecological product is acceptable.	0.930			
A special effort to purchase the green product is acceptable.	0.941			
**Dinning Intention**		0.888	0.930	0.817
I am planning to visit a restaurant featuring organic food in the future.	0.899			
I intend to visit a restaurant featuring organic food in the future.	0.924			
I will expend effort on visiting a restaurant featuring organic food in the future.	0.887			

Table 4 shows that the square root of each AVE is greater than its construct correlations to assess discriminant validity. As a result, the five constructs are relatively independent of one another (Moore and Benbasat, 1991). In Table 5, the HTMT analysis results show that all ratios are lower than 0.85 (Henseler et al., 2015). These findings reflect a good discriminants’ validity of measurement model (Gefen et al., 2000).

**Table 4:** Fornell-Larcker criterion analysis.

**Variables**	**ATO**	**DI**	**EC**	**GEN**
**ATO**	0.897			
**DI**	0.663	0.904		
**EC**	0.594	0.553	0.933	
**GEN**	0.456	0.464	0.519	0.730

GEN: Generativity, ATO: Attitude toward Organic Food, EC: Environment Concerns, DI: Dinning Intention.

**Table 5:** Heterotrait-Monotrait Ratio (HTMT) analysis.

**Variables**	**ATO**	**DI**	**EC**
**ATO**			
**DI**	0.733		
**EC**	0.644	0.610	
**GEN**	0.492	0.511	0.559

GEN: Generativity, ATO: Attitude toward Organic Food, EC: Environment Concerns, DI: Dinning Intention.

### Structure model evaluation

**Figure 2:**
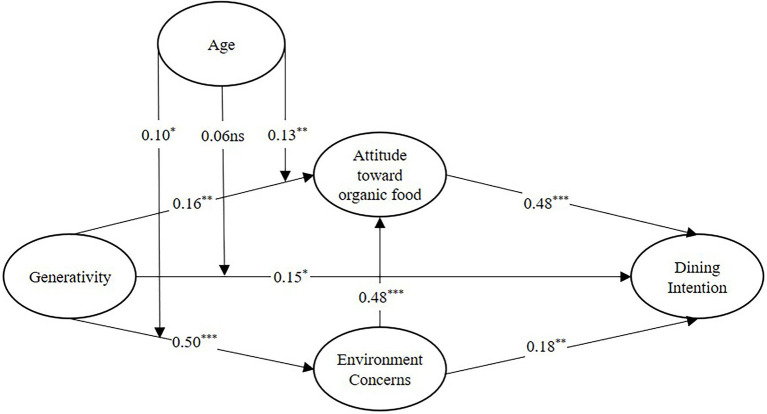
Structural model.

Collinearity was tested using the variance inflation factor (VIF). The findings shows that all VIFs are less than 5, ranging from 1.272 to 1.772, indicating that there are no severe multicollinearity issue in the model (Hair et al., 2011). The 5,000 bootstrapping resampling aims to test hypotheses. The t value of 1.96 was taken as the significant level in the two-tailed test, with 0.05 as the threshold (Hair et al., 2021). Table 6 reveals that the testing result of all hypotheses: H1 (*β* = 0.164, *t* = 3.268, *p* < 0.001), H2 (*β* = 0.477, *t* = 9.635, *p* < 0.001), H3 (*β* = 0.149, *t* = 2.418, *p* < 0.05), H4 (*β* = 0.502, *t* = 9.633, *p* < 0.001), H5 (*β* = 0.475, *t* = 9.663, *p* < 0.001), H6 (*β* = 0.181, *t* = 3.250, *p* < 0.01). The result indicates H1, H2, H3, H4, H5 and H6 are all supported (see Figure 2).

**Table 6:** Direct path for the structural model.

Hypotheses	β	f^2^	*T* Value	*p* Value	Support
H1: GEN→ATO	0.164	0.029	3.268	0.001	Yes
H2: ATO→DI	0.477	0.271	9.635	0.000	Yes
H3: GEN→DI	0.149	0.027	2.418	0.013	Yes
H4: GEN→EC	0.502	0.279	9.633	0.000	Yes
H5: EC→ATO	0.475	0.262	9.663	0.000	Yes
H6: EC→DI	0.181	0.038	3.250	0.001	Yes
H7a: GEN*AGE→ATO	0.093	–	2.538	0.011	Yes
H7b: GEN*AGE→EC	0.134	–	3.058	0.002	Yes
H7c: GEN*AGE→DI	0.059	–	1.623	0.105	No

**Figure 3:**
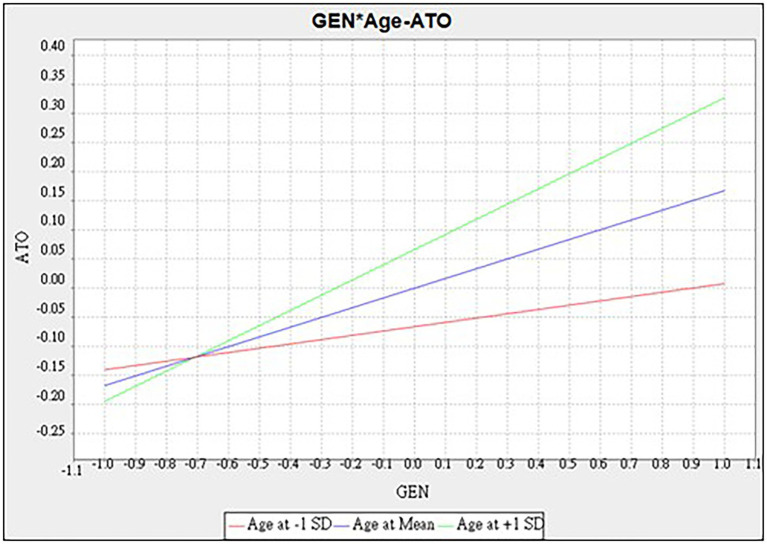
Moderating effect of age-generativity to attitude.

**Figure 4:**
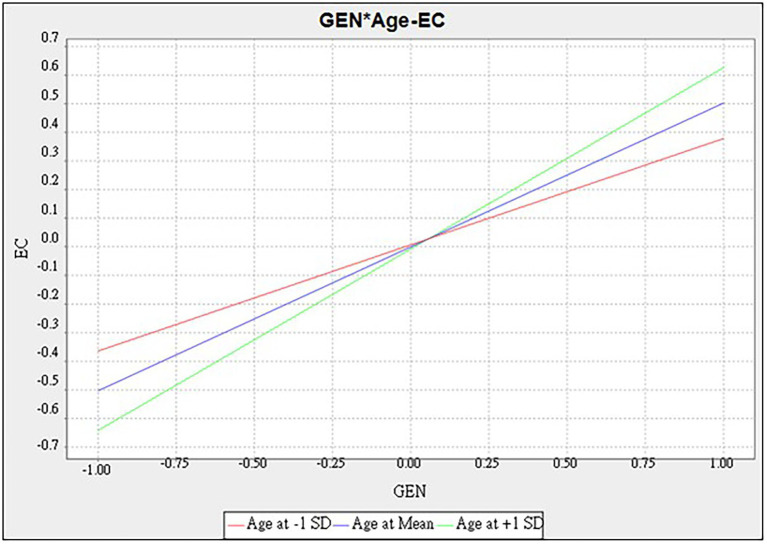
Moderating effect of age-generativity to environmental concerns.

The results of the moderating effect of age are shown in Table 6 and Figures 3, 4. In Figure 3, age positively moderated the causal relationship to attitude toward organic food. The path of generativity to attitude was moderated by age (*β* = 0.093^*^, *t* = 2.538, *p* < 0.05). This means that the older respondents’ generativity shows a higher generativity on attitude toward organic food causality. In Figure 4, the path of generativity to environmental concerns was moderated by age (*β* = 0.134^**^, *t* = 3.058, *p* < 0.01), which meant that older respondents’ generativity shows higher a generativity to environmental concern causality. Older respondents are affected by generativity in their concerns to the environment. However, generativity dose not significantly fuel dining intention in the case of older respondents (*β* = 0.059, *t* = 1.623, *p* > 0.05). Results show that H7a and H7b were supported by the data, while H7c was rejected.

The determination coefficient (R^2^), is frequently used to quantify prediction accuracy (Hair et al., 2021). The result presents the R^2^ value between 0.283 and 0.492 are acceptable (Table 7). The *f* ^2^ effect size quantifies the actual impact of the anticipated variable (see Table 5). These data meet the criteria for indicators greater than 0.02. A predictive correlation (Q^2^) larger than zero indicates that the prospective variable has predictive correlation for a certain dimension (Chin, 1998; Henseler et al., 2009). Table 7 also reported that all Q^2^ assessment results are larger than zero, indicating that the structural model in this study has an adequate predictive capacity.

**Table 7:** R2 and Q2.

Variables	R2	Q2
**ATO**	0.388	0.311
**DI**	0.492	0.399
**EC**	0.283	0.245

The bootstrapping resampling approach was also used to investigate the mediation effect of environmental concerns and attitudes toward organic food. The interval is less than 0.05, indicating that the mediation effect is valid (Table 8). This result indicates that generativity affected dining intention through environmental concerns and attitude.

**Table 8:** Result of the mediation tests.

Path	β	*T* Value	*p* Value	Support
GEN→EC→DI	0.091	3.060	0.002	Yes
GEN→ATO→DI	0.078	3.143	0.002	Yes
GEN→EC→ATO→DI	0.114	5.777	0.000	Yes
GEN→EC→ATO	0.238	7.182	0.000	Yes
EC→ATO→DI	0.226	6.714	0.000	Yes

## Discussion

This study investigated the consumers’ generativity, attitudes, environmental concerns and organic dining intention. By linking the present and the future in the context of environmental issues, it helps to understand the antecedents and consequences of consumers’ behavioral intentions toward organic restaurants. It was found that this complex phenomenon comprises the integration of various variables and that the extent to which generativity influences organic restaurant intentions depends on various intervening mechanisms. This study verified the direct and indirect effects between variables to reveal their relationships. On the structural model, results show that generativity directly affects the consumers’ attitudes toward organic food (H1), and their attitude significantly influence intention to eat (H2). Consumer’s recognition of organic food as healthy and environment friendly prompts their positive attitudes that lead to consumption behavior. Since consumers’ attitudes toward organic food positively influence their dining intention, enhancing consumers’ attitudes toward attributes of organic food, such as price, tastes, appearance, and environmental benefits, is essential for promoting dining behavior (Bryła, 2016). Furthermore, this study confirms that generativity positively influences consumers’ dining intention (H3), so operators should attach importance to the environmental impact of producing organic food to establish a link between consumers’ generativity of sustainable development and organic food consumption. Attitudes positively and significantly mediated the consumers’ generativity and dining intention in this study. This indicates that people with higher generativity are more likely to have positive attitudes and dining intentions. The current study validated the Value-Attitude-Behavior model with generativity value as the antecedent variable.

This study found that generativity may positively influence environmental concerns (H4), wherein the result is consistent with previous studies (Pratt et al., 2013). Meanwhile, environmental concerns positively affect the attitude toward organic food (H5). The current study revealed that consumer environmental concerns can enhance their positive attitude toward organic food, which is highly expected in a green marketing sector (Khaola et al., 2014). The current study unveiled that environmental concerns could result in consumers’ dining intention (H6). However, the result refutes the research by Cleveland et al. (2005), in which environmental concerns do not directly influence green purchase behavior. Moreover, our findings indicate that environmental concerns positively and significantly mediate the relationship between generativity and dining intention. This result contradicts Malik et al. (2019). The present study confirms that individual with higher generativity values, who are more concerned about future generations, show higher environmental concerns and lead to environmentally friendly behaviors.

Furthermore, in this study, age plays a positive moderating role in the relationship between the influence of generativity on environmental concerns and attitude, respectively. The result indicated that older individuals are more likely to be affected by generativity on attitude toward organic food (H7a). Meanwhile, the older individuals are more likely to be influenced by generativity on environmental concerns (H7b). However, age play a positive but nonsignificant moderating role in the relationship between generativity and dining intention (H7c). This may explain that different age groups have different consumption habits and different decision-making methods. Prior studies have proved that the food consumption patterns were not uniform among different age groups (Abdel-Ghany and Sharpe, 1997; Vatanparast et al., 2019). Therefore, despite higher generativity may lead to higher organic dining intention. This relationship might not be influenced by consumer’s age due to different food consumption patterns among age groups. Another issue proven by the results of this study is that the generativity is more likely to manifest itself in an older age group. The older they are, the more they are concerned about environmental issues, so they have a more positive attitude toward organic food.

The results of the present study show that respondents perceive themselves as a valuable role for future generations are more indulged in behavioral intentions of organic restaurants. This indicates that those who perceive their expected contribution to be significant are more inclined to choose organic food. The findings consistent with previous literature (Shiel et al., 2020). This study focuses on the process of generativity influencing behavioral intentions in organic restaurants. The concept of generativity originated from Erikson (1950a), and was introduced into studies of environmental protection behavior in the Western society context (Pentland and Feldman, 2005; Henfridsson and Bygstad, 2013; Cennamo and Santaló, 2019). To a certain extent, this study adds knowledge by discussing it in the context of Eastern society. These results convey key messages for the restaurant industry, especially for restaurants which feature organic food, determining the success of the restaurant. Our study indicates that generativity helps consumers in positively evaluating the behavior of visiting organic restaurants.

## Conclusion

### Theory implications

The current study provides remarkable contributions to generativity and organic restaurant intention behavior literature. This study developed a research framework for green purchase behavior to explain the understanding of organic restaurant customer behavior. This study introduces the generativity in the field of consumer restaurant intentions and validates the VAB model with generativity as the antecedent variable. Generativity had a positive relationship with attitude, consistent with the results of prior studies (Lacroix and Jolibert, 2017; Afridi Ahmad et al., 2021b). Theoretically, this study adds to the literature on the psychosocial attributes of generativity on green purchase behavior in the hospitality industry. This study examined the positive effects of generativity on organic food attitudes, environment concerns and dining intention.

Moreover, this study validation of environment concerns mediation role in the framework. This is another significant contribution in this study. According to the findings of the current study, generativity has a favorable impact on environmental concerns. Individuals with higher generativity may be able to satisfy their generative needs by being concerned about environmental issues (Sharma et al., 2022), which lead pro-environment purchase behavior. In addition, the positive relationship between environment concerns and organic dining intention, is consistent with previous literature (Shiel et al., 2020). The results of this study contribute to a better understanding of how consumers are influenced by their intrinsic generativity values affecting environmental concerns and attitudes in the organic restaurant consumption environment, providing insight into new green behaviors.

Finally, the current study separately verified the positive moderating effect of age in generativity on the attitude and environment concerns. The results of this study fill the research gaps because prior studies have not examined the moderating effect of age in generativity on the attitude and environment concerns in green purchase context. Therefore, the current study added to the existing knowledge by exploring and understanding the role of generativity and the affected environment concerns, as well as attitude in predicting dining intention.

### Practical implications

The theoretical framework of this study provides elements which may influence organic restaurant intentions and contribute practitioners to understand the antecedents of consumer green purchase behavioral intentions. Practical recommendations are provided for professionals in the hospitality and marketing departments. Based on the results of the study, practitioners can play the role of consumers’ generativity through building the connections between organic food production and the ecological environment of future generations. In turn, the consumer will be more willing to consume in organic restaurants can stimulate sales. For example, campaigns could emphasize children’s and grandchildren’s future through the lens of a greener world and better living circumstances in the future. By providing this support, restaurant practitioners can influence consumers’ concerns about the environment and the importance of sustainability for future generations, promoting positive attitudes toward organic food and behavioral intentions toward organic restaurants. This strategy will attract not only current consumers, but also new customers whose beliefs are closely linked with supporters of environmental protection. Moreover, the findings also revealed the significant role of consumers’ attitudes toward organic food. Compared with alternative food options, the results imply that restaurant managers should focus on changing customer’s attitudes toward attributes of organic food, such as price, variety, appearance, and taste.

Notably, our results show that the effect of generativity on environmental concerns and attitudes toward organic food is more among older age groups customers. As demonstrated in this study, the relationships that affect environmental concerns and attitude toward organic food improve substantially in older ages. The design of restaurant menus could be more focused on catering to the taste habits of older ages. We can strengthen the environmental awareness of young people through green propaganda and increase their acceptance of organic food. Restaurant operators train their restaurant servers on communication strategies regarding the green practices used in their restaurants, wherein the restaurant’s business philosophy can be more effectively communicated to consumers.

### Limitations and future research

There are some limitations in this study. Firstly, our sample was limited to Shanghai, while consumers in the same city may have similar levels of and cultural backgrounds. Future studies should target consumers from various cultural backgrounds, especially those related to green restaurants. For example, sampling from respondents from various countries or geographic regions. Secondly, this study adopted a non-probability and cross-sectional sampling method, which may reduce the representativeness of samples and not warrant causal inference. Thirdly, consumer purchase behavior is often complex, and this study only considered two mediating variables, perhaps other variables, such as corporate social responsibility, pricing, and trust, may also be relevant in explaining this process. Finally, this study used behavioral intentions to predict consumption behavior; future studies should include actual consumption behavior to help obtain an objective assessment when predicting consumption behavior.

## Data availability statement

The raw data supporting the conclusions of this article will be made available by the authors, without undue reservation.

## Author contributions

JL and YP: conceptualization and validation. YP: methodology, software, formal analysis, investigation, resources, data curation, visualization, project administration, and writing—original draft preparation. YP and JX: writing—review and editing. JL: supervision. All authors contributed to the article and approved the submitted version.

## Conflict of interest

The authors declare that the research was conducted in the absence of any commercial or financial relationships that could be construed as a potential conflict of interest.

## Publisher’s note

All claims expressed in this article are solely those of the authors and do not necessarily represent those of their affiliated organizations, or those of the publisher, the editors and the reviewers. Any product that may be evaluated in this article, or claim that may be made by its manufacturer, is not guaranteed or endorsed by the publisher.
